# Eucommia, Cuscuta, and Drynaria Extracts Ameliorate Glucocorticoid-Induced Osteoporosis by Inhibiting Osteoclastogenesis Through PI3K/Akt Pathway

**DOI:** 10.3389/fphar.2021.772944

**Published:** 2022-02-04

**Authors:** Junwen Han, Li Li, Chen Zhang, Qianqian Huang, Shanglong Wang, Wenyu Li, Jiancheng Zong, Lijie Li, Zhen Zhao, Zengliang Zhang, Zimin Liu, Qi Wang, Yuanyuan Shi

**Affiliations:** ^1^ College of Traditional Chinese Medicine, Beijing University of Chinese Medicine, Beijing, China; ^2^ Chenland Nutritionals, Inc., Irvine, CA, United States; ^3^ School of Life Sciences, Beijing University of Chinese Medicine, Beijing, China; ^4^ Beijing University of Chinese Medicine Affiliated Third Hospital, Beijing, China; ^5^ Qingdao Engineering Vocational College Dongzhang Community, Qingdao, China; ^6^ Pathology and Laboratory Medicine, Weill Cornell Medical College, New York, NY, United States; ^7^ Traditional Chinese Medicine College, Inner Mongolia Medicine University, Hohhot, China; ^8^ National Institute of Chinese Constitution and Preventive Medicine, Beijing, China; ^9^ Shenzhen Research Institute, Beijing University of Chinese Medicine, Shenzhen, China

**Keywords:** Eucommia, Cuscuta, Drynaria, Traditional Chinese medicine, Osteoporosis, Osteoclast, PI3K/Akt

## Abstract

Osteoporosis is one of the most common diseases in the world which resulted in heavy socioeconomic burden and a public health threat. Glucocorticoid-induced osteoporosis (GIO) is the most common secondary reason of osteoporosis. Therapeutic strategies using traditional Chinese medicine are under investigation for osteoporosis, with efforts to improve efficacy and clarify the mechanism. The combination of Eucommia, Cuscuta, and Drynaria is widely used in traditional Chinese decoction for osteoporosis treatment, but the experimental efficacy and mechanism are still unclear. Administration of E.C.D. extracts (Eucommia, Cuscuta, and Drynaria) in experimental GIO rats resulted in decreased urinal calcium, phosphorus loss, and decreased expression of RANKL, CTX in serum, increased serum calcium, phosphorus, and OPG level. E.C.D. extracts also improved bone density, structural integrity, and biomechanical function in experimental GIO rats. These finding were associated with E.C.D. extracts’ treatment efficacy to GIO *in vivo*. The balance between osteoclast and osteoblast activity is essential for bone remodeling and bone related disease. The E.C.D. extracts inhibited Raw 264.7 cell differentiation to osteoclast *in vitro*. On the other hand, it promoted OPG expression of bone marrow mesenchymal stromal cells (MSCs) which can suppress the osteoclast genesis. E.C.D. extracts also increased the Wnt1 and Runx2 expression which are related to osteoblast formation. It also regulated the paracrine effect of MSC to inhibit osteoclast differentiation. The analysis of HPLC and comprehensive pharmacology identified the constituents of E.C.D. extracts and the potential osteoporosis-related targets mediated by E.C.D. extracts. The KEGG enrichment analysis suggested that PI3K/Akt pathway may be involved in the regulation osteoclast genesis by E.C.D. extracts and the result of Western blot of *vitro* assays proved it. Collectively, these data demonstrate E.C.D. extracts can inhibit osteoclast differentiation to foster experimental osteoporosis both *in vivo* and *in vitro* and it may exert the function of inhibiting osteoclast differentiation through PI3K/Akt pathway.

## Introduction

Bone remodeling is a dynamic process in which activated osteoclasts resorb bone and osteoblasts generate a bone matrix that undergoes mineralization (Sözen et al., 2017). This biological process repairs micro-damages that develop in bone during daily activity and protects skeletal strength (Sambrook and Cooper, 2006). When the balance of bone remodeling is disrupted, bone resorption is greater than bone formation, resulting in osteoporosis. Glucocorticoids (GC) are widely used in clinic, but they also bring serious side effects, one of which is glucocorticoid-induced osteoporosis (GIO) (Long, 2020). GIO is the most common secondary reason of osteoporosis. Fractures could occur in as many as 30–50% of patients who have received chronic glucocorticoid therapy (Canalis et al., 2007). The direct mechanism of GIO is upregulating PPARγR2 and RANKL expression and decreasing OPG expression, which results in bone resorption (Compston, 2018).

As more mechanisms of osteoporosis were discovered and novel techniques had been developed to help better understand about the micro-structure of bone, there are several effective therapies for osteoporosis treatment including bisphosphonates, elective estrogen, and RANKL inhibitor such as denosumab (Khosla and Hofbauer, 2017; Choksi et al., 2018), as well as teriparatide (Chotiyarnwong and McCloskey, 2020). Despite multiple therapies having been applied to clinical treatment, there are still high fracture risks and side effects of patients who have osteoporosis (Khosla and Hofbauer, 2017). The side effects and long-term effects of the novel drugs drive the development of new therapy.

The structure of bone is made of three parts: cells (osteocytes, osteoblasts, and osteoclasts), organic components (matrix proteins), and nonorganic components (calcium and hydroxyapatite) (Buehring et al., 2013). Bone’s metabolism balance largely depends on the activity of osteoclast and osteoblast. Osteoclast origins from hematopoietic mononuclear cell while osteoblast origins from bone marrow mesenchymal stromal cell (MSC) (Chen et al., 2016; Andrzejewska et al., 2019). The classic pathway published before is RANKL/OPG pathway which regulates osteoclast differentiation and osteoblast genesis (Silva and Branco, 2011; Tobeiha et al., 2020). However, the upstream targets and other proteins are gradually discovered playing important roles in bone metabolism (Armas and Recker, 2012). The phosphatidylinositol 3-kinase (PI3K)/Akt signaling pathway regulates a broad range of biological processes including cell proliferation, growth, and anti-apoptosis (Ersahin et al., 2015). PI3K/Akt signaling pathway also has been shown to regulate osteoclast differentiation (Yeon et al., 2019; Cui et al., 2020). However, the downstream proteins which can be regulated by PI3K/Akt are abundant and not clearly clarified. Thus, to find the specific pathway that regulates the bone metabolism is important.

Eucommia, Cuscuta, and Drynaria (E.C.D.) are widely used as a combination and play a synergy in traditional Chinese medicine (TCM) decoction for osteoporosis treatment (Jia et al., 2012; He et al., 2019). The combination of E.C.D. is an important component in many decoctions and prescriptions in orthopedics such as Zhuang Jin Xu Gu Dan (<Shang Ke Da Cheng>, Qing Dynasty), Tu Si Zi Wan, etc. The combination of E.C.D. is also published to treat weakness of the feet and knees (Sang Shurong, 1993). <Zheng Zhi Zhun Sheng • Yang Yi> (Volume 2, Ming Dynasty) records the Drynaria pill using the combination of E.C.D. to treat flaccidity and arthralgia. <Bai Yi Xuan Fang> (Song Dynasty) use the combination of Eucommia and Cuscuta to treat low back pain. For bio-pharmacology study, Eucommia extracts were published to alleviate osteoporosis in rats and regulate osteoblast differentiation through the Shp2/PI3K/Akt pathway (Zhou et al., 2016). Mo H. et al. reported Cuscuta can modulate RANKL/OPG (Mo et al., 2019). Drynaria was proved to suppress the RANKL signal to inhibit osteoclast differentiation (Lin et al., 2013). The combination of E.C.D. has been developed as an adjunct to dietary supplements for treating osteoporosis (EuBone, Batch No.BH201-20201013-01, Product code: T-4005-1). It is also applying for a patent of novel formula to promote bone repair (patent application number: 2020105750230). Even though there is a lot of evidence of therapeutic application of the combination of E.C.D. in osteoporosis treatment, the efficacy of the combination of E.C.D. in experimental models and the further mechanism are still not clearly clarified. Thus, we proposed that E.C.D. extracts can alleviate experimental osteoporosis by regulating bone metabolism balance.

## Materials and Methods

### Preparation of E.C.D. Extracts

Eucommia (*Eucommia ulmoides Oliv.*) was purchased from Anhui Zhiliang Traditional Chinese Medicine Decoction Pieces Co., LTD. Cuscuta (*Cuscuta chinensis Lam*) and Drynaria (*Davallia trichomanoides Blume*) were purchased from Bozhou Traditional Chinese Medicine Commodity Trading Center Co., LTD. Raw medicinal materials were identified by HPLC according to the method developed by Alkemist Las IDTSOP-72-01 in the U.S (Supplementary Figure S1). The E.C.D. extracts were made from ground Eucommia, Cuscuta, and Drynaria at a ratio of 6:1:3 (the ratio is based on the calculation of pharmacopoeia recommended dosage and extracts rate) into powder and sieved through a 20-mesh sieve. The extraction process of counter-current extraction (CCE) was based on a previous publication (He et al., 2017). Briefly, 5 L of 95% ethanol was added to 500 g of dried powder. Then, the mixture was extracted twice at room temperature (each time was 60 min). The extract was then filtered and condensed under vacuum, and then further dried using a freeze-dryer. For *vitro* delivery, E.C.D. extracts were dissolved in DMSO solution (100 mg/ml) and sonicated for 1 h in room temperature then centrifuged 3,000 rpm for 10 min. The supernatant was taken and diluted in DMEM medium in different doses for further experiment.

### Animals

A total of 40 2-month-old female Wistar rats, weight 200 ± 20 g, were used in the present study. Rats were housed in room temperature (25°C) with 60% humidity and a 12-h light/dark cycle in the Laboratory Animal Department (4 rats per cage). After adaptation for 1 week, rats were randomly divided into five groups (*n* = 8 per group). The rats in the Vehicle group received saline intramuscular injection twice/week and saline by gavage 7 days/week. Other rats were proceeded of prednisolone hydride (4.5 mg/kg) intramuscular injection twice/week to induce GIO. After GC administration rats received either saline (GC group) or E.C.D. extracts of 90 mg/kg/day (E.C.D.-L group), or E.C.D. extracts of 135 mg/kg/day (E.C.D.-M group), or E.C.D. extracts of 180 mg/kg/day (E.C.D.-H group) by gavage for 7 day/week. The body weight and temperature of each rat was measured every week. 8 weeks later, rats were placed in a metabolic cage to collect overnight urine. The 24-h urine of rat was centrifuged at 3,500 rpm/min 4ºC for 15 min, the supernatant was separated, and stored in a −80°C refrigerator for further test. The rats were then scarified, and the blood was collected from each rat. Collected blood was centrifuged at 2,000 rpm for 20 min to collect serum. Femurs of two sides were also collected and filled in saline and stored at −20°C for measurement of bone mineral content (BMC), bone mineral density (BMD), trabecular microarchitecture by micro-CT, and bone biomechanical quality by a three-point bending test.

### Cytokine Analysis of Serum and Urine

Serum calcium and phosphorus concentration were measured by Siemens ADVIA 2400 blood biochemical analyzer. Urine calcium and phosphorus concentration were analyzed by standard colorimetric methods using commercial kits (ZhongSheng BeiKong Biotechnology and Science, PRC) and analyzed by a Cobas Integra 400 Plus automatic biochemical analyzer (Roche Diagnostics, Switzerland). Serum Osteocalcin, CTX, OPG, and RANKL concentration were determined using rat ELISA kit (R&D, United States; for details, see Supplementary Table S2).

### Histology and H&E Staining

Rats were sacrificed 8 weeks following GC or saline administration. After a biomechanical test, the right femur was processed in 10% EDTA for bone demineralization of 8 weeks. The EDTA decalcified liquid was replaced every 3 days. Then the bone was fixed in 10% formalin, processed, embedded in paraffin, and sectioned (5 µm). Bone sections were assessed by hematoxylin and eosin (H&E) stain (abcam, Catalog number: ab245880) following the company’s manuscript. The TRAP staining was performed using a Trap Stain Kit (Servicebio, Catalog number: G1050). The images were observed by Olympus BX51 light microscopy (Olympus, Japan).

### Biomechanical Testing

The attached muscle and connective tissue of the right femur were removed. The right femur then was subjected to the 3-point bending test using a Lloyd material testing machine (LR5K; J.J. Lloyd Instruments, Southampton, United Kingdom). The femora specimens were placed on their lateral surface on the two supports of the bending apparatus. The bending load was applied at a rate of 1.0 mm/s (span 15 mm, transverse distance 2–3 mm) on the midpoint of the femora diaphysis until failure of the bone specimen. The breaking strength indexes including maximal load (Fmax) and elastic load (Fp) were measured.

### Microtomographic Histomorphometry by Micro-CT

The left femur was thoroughly dissected from soft tissue and then was fixed with 4% paraformaldehyde for 24 h, and subsequently washed with 10% saccharose solution. A total of 12 h later, microtomography of the proximal femur was performed by micro-CT camera scanner (Bruker, Skyscan1174). Three-dimensional images of each proximal femur were acquired with a voxel size of 10.34 μm in all spatial directions. A cone-beam-type desktop micro-CT system was used to quantify structural parameters of the femur. The analytical conditions were 80 kV with 800 μA, and the spatial resolution was 14 μm according to the protocol of largetube-14 μm-150 min-SS for about 500 consecutives sections. For all femurs, a cylinder region of interest (ROI, diameter 3 mm × height 4 mm) was chosen to reconstruct three-dimensional images using Micview software. After thresholding, trabecular thickness (Tb.Th), trabecular number (Tb.N), trabecular separation (Tb.Sp), bone volume/total volume (BV/TV), structural model index (SMI), and bone mineral density (BMD) were determined.

### Liquid Chromatography Mass Spectrometry Analysis

We performed HPLC analyses for the identification of chemical fingerprints presented in E.C.D. extracts. Briefly, the E.C.D. extracts were dissolved in methanol (1 g/ml) and then sonicated for 1 h. The extracts-methanol mixture was centrifuged at 12,000 rpm for 10 min and supernatant was taken and then diluted 1:10 with methanol. The sample was ready for HPLC analysis.

The Dionex Ultimate 3000 U. UPLC system was made up of LTQ-Orbitrap XL (Thermo Fisher Scientific, San Jose, CA) controlled by Xcalibur software (Version 2.1), a binary pump, an autosampler, a solvent degasser, and a thermostatic column compartment (Thermo Fisher Scientific, United States) were used for analysis component of CuraUltrad. Mass spectrometer (MS) detection was performed on a Q Exactive™ Plus mass spectrometer (Thermo Fisher Scientific, United States) equipped with an electrospray ionization source (ESI).

All chromatographic separations were performed on a Waters Acquity UPLC BDS C18 column (150 mm × 2.4 mm, 2.74 μm). The column temperature was 35°C, the flow rate was 0 ml/min. The mobile phase consisted of acetonitrile (A) and 0.1% formic acid aqueous solution (B) at a flow rate of 0.3 ml/min, with gradient elution as follows: 0– min, 95% B; 3– min, 95%–25% B; 45–45. min, 25%–95% B; 45.1–50 min, 95% B.

In negative and positive ion modes, the mass conditions were set. Briefly, the gas of sheath and auxiliary was nitrogen. The flow rates were 40 arbitrary units and 20 arbitrary units, respectively. The capillary temperature was 320°C. The sheath gas flow rate was 35.0 μL/min. The aux gas flow rate was 10 μL/min. The sweep gas flow rate was 10 μL/min. The positive ion spray voltage was 3.5 KV while the negative ion spray voltage was 3.0 KV. In addition, the mass scanning range was m/z 120-1800.

### The Analysis of Constituents of E.C.D. Extracts

The information of published constituents of Eucommia, Cuscuta, and Drynaria was collected based on TCMSP database (https://old.tcmsp-e.com/tcmsp.php). The molecular 2D files of these constituents were downloaded from the ChemSpider database (http://www.chemspider.com/) and were saved in mol format. The mass spectra of E.C.D. extracts were recorded across the range of m/z 120-1800. All the data were processed using Xcalibur software version 2.7. The constituents of E.C.D. extracts found in mass spectra were compared with published information and the TCMSP database then the molecular information was recorded in Supplementary Data Sheet S1.

### The Prediction of E.C.D. Extracts Related Targets

The predicted proteins targeted by E.C.D. extracts were screened using MedChem Studio software based on the mass spectrometry analysis. Briefly, the identified constituents of E.C.D. were input into the SwissTargetPrediction database (http://www.swisstargetprediction.ch/). We picked the targets of ingredients of E.C.D. with high confidence score (≥1) and recorded the target information.

### Network Construction and Analysis

Known therapeutic targets for osteoporosis were collected from GeneCards database (http://www.genecards.org/), OMIM database (https://www.omim.org/). and DrugBank database (http://www.drugbank.ca/). After integrating data from several databases and removing duplicate data, we took the intersection of the verified constituents target of E.C.D. based on previous analysis and the therapeutic targets of osteoporosis. The constituents–targets network was constructed using Cytoscape software (Verson 3.8.2, Boston, MA) and Java analysis.

### Pathway Enrichment Analysis

Kyoto Encyclopedia of Genes and Genomes (KEGG) pathways analysis was undertaken to explore the potential functions of the pivotal target proteins involved in the E.C.D extracts-mediated treatment of osteoporosis using DAVID Bioinformatics Resources 6.8 (https://david.abcc.ncifcrf.gov/home.jsp). Relevant pathways with the false discovery rate (FDR)-corrected *p*-value < 0.05 were considered statistically significant. Other parameters were set as the default values. Meanwhile, the biological process (BP), cellular components (CC), and molecular function (MF) of target protein involved were also analyzed.

### Cells

The murine Raw264.7 cell line was purchased from Xie He Hospital, Beijing, China. The cells were cultured in DMEM medium (Geibo, Catalog number: 11,995-065) with 10% fetal bovine serum (FBS) and 1% penicillin/streptomycin/L-glutamine (P.S.G.). The cells of passage 11-15 were used in the experiment. The murine bone marrow MSC was harvested from C57BL/6 male mice, 6 weeks of age. Briefly, the mice were sacrificed by broken vertebral column. The tibia and femur were isolated and then washed by PBS. The bone was crushed and washed 4 times with PBS. Collection of PBS was centrifuged at 1500 rpm for 10 min. Then 0.1% Collagenase I (Worthington, Catalog number: LS004194) and 0.25% Collagenase II (Worthington, Catalog number: LS004174) were added to bone fragments and the mixture was incubated in a 37 ºC shaker for 1 h. Supernatant was collected and then centrifuged in 1500 rpm for 10 min. The cells from two steps were seeded in 100 mm dish for 3 days. MSC was cultured using MesenCult™ Expansion Kit (Mouse) (STEMCELL, Catalog number: 05513). MesenPure™ (STEMCELL, Catalog number: 05510) was used to keep MSC homogeneous and maintain robust proliferation, differentiation, and colony formation capacity. Passage 4-6 of MSC was used in this experiment.

### CCK8 Assay

The Cell Counting Kit-8 (Biorigin, Catalog number: BN15201) was used for this experiment. Basically, Raw264.7 cells or MSCs were seeded in 96-well plate overnight. Then E.C.D. extracts of different doses were added to each well and co-incubated for 24 h or 48 h. Then the medium was replaced and 10 μL CCK was added to each well maintained at 37°C in a humidified 5% CO_2_ atmosphere for 1 h. The OD number was read in 450 nm.

### Osteoclast Differentiation Assay

RAW 264.7 cells were cultured at a density of 2 × 10^5^ cells/well in 6-well plates in the presence of 50 ng/ml recombinant mouse RANKL (Novoprotein, Catalog number: CR06, China) and 50 ng/ml recombinant mouse M-CSF (Novoprotein, Catalog number: CB34, China). M-CSF and RANKL (C/R) were replenished every 2 days. Cultures were maintained at 37°C in a humidified 5% CO_2_ atmosphere. At the same time, E.C.D. extracts (1 μg/ml, 50 μg/ml, 200 μg/ml) or MSC CM or E.C.D. pre-treated MSC CM were added for 48 h, 96 h, or 5 days.

### RNA Extraction and qPCR

The E.C.D. extracts were added to Raw264.7 cells or mouse bone marrow derived MSCs at different doses (1 μg/ml, 50 μg/ml, 200 μg/ml). RNA was harvested after 48 h incubation. The RNA was extracted with Trizol Reagent (QIAzol, Catalog number: 79306). Reverse transcription (RT) was conducted using Evo M-MLV RT kit with gDNA Clean for qPCR II (Accurate Biology, Catalog number: AG11711). Real-time amplification of complementary cDNA was conducted in a Rotor-Gene 3000 System (Corbett Research, Mortlake, Australia) with the 2X Realab Green PCR Fast mixture (Universal) (LABLEAD, Catalog number: R0202-03). The primers of mouse used are as follows:

NFATc1, 5′-TCATCCT GTCCAACACCAAA-3′(Forward) and 5′-TCA​CCC​TGG​TGT​TCT​TCC TC-3′(Reverse); TRAP, 5′-CAGCAGCC AAGGAGGACTAC-3′(Forward) and 5′-ACA​TAG​CCC​ACA​CCG​TTC TC-3′(Reverse); RANKL, 5′-TGT​ACT​TTC​GAG​CGC​AGA​TG-3′ (Forward) and 5′-AGG​CTT​GTT​TCA​TCC​TCC​TG-3′ (Reverse); Runx2, 5′-CGA CAG TCC CAA CTT CCT GT-3′ (Forward) and 5′-CGG TAA CCA CAG TCC CAT CT-3′(Reverse); Wnt1, 5′-CTTCGGCAAGATCGTCAAC-3′(Forward) and 5′-CTGCCTCGTTGTTGTGAAG-3′(Reverse); OPG, 5′-ACA​GTT​TGC​CTG​GGA​CCA​AA-3′ (Forward) and 5′-TC ACAGAGGTCAATGTCTTGGA-3′(Reverse).

### Protein Extraction and Western Blot

The E.C.D. extracts were added to Raw264.7 cells with C/R (50 ng/ml) stimulation at different dose (1 μg/ml, 50 μg/ml, 200 μg/ml). After 48 h or 96 h, the protein was harvested by RIPA buffer (APPPLYGEN, Catalog number: C1055) with protease inhibitor (10X) and phosphatase inhibitor (10X). The protein concentration was measured by BCA assay (APPPLYGEN, Catalog number: P1511) following the company’s manual. After adding loading buffer (5×), the lysed protein was boiled at 100°C for 10 min and then equal protein concentration was electrophoresed on 8 or 12% gels. Antibodies for blotting (NFATc1, RANKL, TRAP, PI3K, p-Akt, Akt, p-P38, P38, GAPDH) are detailed in Supplementary Table S1. Protein expression was assessed using ImageJ software.

### Tartrate-Resistant Acid Phosphate Staining Assay

The Raw264.7 cells were cultured in 96-well plate at the density of 5X10^3 cells/well. The C/R of 50 ng/ml were added for 5 days, and medium was changed every other day. E.C.D. extracts of different concentrations were added in the meantime. Then the TRAP staining was performed following the company’s manual (Servicebio, Catalog number: G1050). Briefly, cells were fixed by 4% paraformaldehyde then permeabilized by 0.2% Triton X-100. The TRAP working solution was added and then incubated in 37°C for 2 h. The TRAP staining was observed under light microscope and the number of TRAP positive cells with ≥ 3 nuclei was counted.

### F-Actin Assay

The Raw264.7 cells were cultured in 35 mm dish at the density of 1X10^5 cells/dish. The C/R of 50 ng/ml were added for 5 days and E.C.D. extracts were added at the same time. Five days later, the F-actin staining was performed using Rhodamine Phalloidin Reagent (abcam, Catalog number: ab235138) as suggested by the manufacturer. Basically, the cells were fixed by 4% formaldehyde at room temperature for 30 min and then permeabilized by 0.1% Triton X-100 for 5 min 1X Phalloidin conjugate working solution was added and incubated at room temperature for 90 min. The DAPI was added for 10 min to stain nuclei. After adding mounting media, the cells were observed using Olympus FV3000 confocal microscope fitted with a filter at Ex/Em = 546/575 nm.

### Bone Resorption Assay

The bovine bone slices (0.4 mm) were put in a 96-well plate, and the Raw264.7 cells were cultured on the bone slice at the density of 1X10^3 cells/well overnight. The C/R of 50 ng/ml were added for 7 days and E.C.D. extracts were added at the same time. The medium was changed every 3 days. After 7 days, the cells were washed off with 10% sodium hypochlorite for 5 min and washed 3 times with distilled water. The bone resorption pit was observed under the light microscope. Then the bone slices were stained using 1% toluidine blue at room temperature for 30 min and washed 3 times with distilled water. The resorption pit was photographed under the light microscope and the percentage of resorption area was assessed using ImageJ software (Fu et al., 2013; Sapkota et al., 2015; Zeng et al., 2016; Liao et al., 2021).

### PI3K Inhibition Assay

The PI3K inhibitor (LY294002) (Kao et al., 2005; Zhao et al., 2015) was purchased from Apexbio (Catalog number: LY294002). LY294002 was added to Raw264.7 cells to reach the final concentration of 30 μM. After 2 h, LY294002 was removed and then C/R of 50 ng/ml were added for another 48 h. Then the protein was harvested and assessed by Western blot.

### Preparation of MSC Conditioned Medium

Mouse bone marrow MSCs from P5-P6 were cultured to 80–90% confluence. The E.C.D. extracts (1 μg/ml, 50 μg/ml, 200 μg/ml) or PBS (same volume) were added to medium for 48 h. The pre-treated MSCs were washed with PBS, then replenished with supplement-free DMEM medium. After 24 h, the CM was collected, and centrifuged at 2000 rpm for 5 min to remove cell debris. The CM was then kept at −80°C until use.

### Statistical Analysis

For comparisons between two groups, we used Student’s unpaired *t* test. For analysis of more than two groups, one-way or two-way analysis of variance (ANOVA) was performed. When data were not normally distributed, non-parametric analyses were performed using Kruskal-Wallis testing. Comparisons of weight change was made by analyzing the area of curves, then two-way ANOVA was performed to assess for differences in weight changes. Statistical significance was accepted at *p* < 0.05.

## Results

### E.C.D. Extracts Protected Body Weight Loss, Calcium and Phosphate Loss of Experimental GIO Rats

**FIGURE 1:**
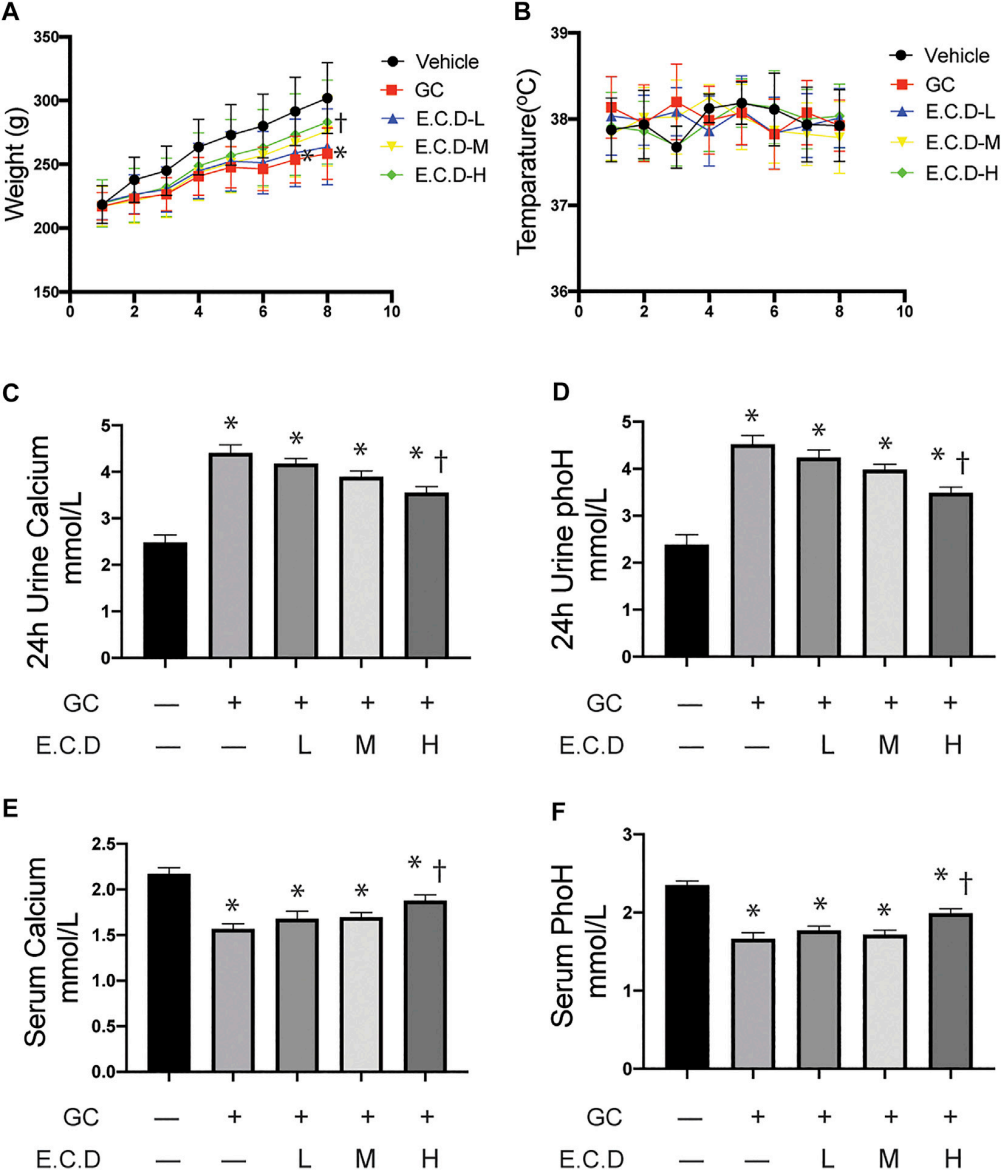
E.C.D. extracts protected body weight loss, and calcium and phosphorus loss of GIO rats. **(A)** Rats were randomly divided into five group, *n* = 8 in each group: Vehicle group (black, dot), GC group (red, square), E.C.D.-L group (blue, triangle), E.C.D.-M group (yellow, down triangle), E.C.D.-H group (green, diamond). The change of body weight from Week 0 to Week 8 was recorded. * GC group vs. Vehicle group, † E.C.D.-H group vs. GC group, *p* < 0.05. **(B)** The temperature of rats was recorded every week (0-8) with no significant difference between each group. **(C,D)** The 24-h urine calcium and phosphorus (phoH) were collected 8 weeks after GC administration, *n* = 8 in each group. **(E,F)** The serum calcium and phosphorus (phoH) were measured 8 weeks after GC administration, *n* = 8 in each group. Data was analyzed as mean ± SEM, * vs. Vehicle group (GC–E.C.D.–), † vs. GC group (GC + E.C.D. –), *p* < 0.05.

Osteoporosis usually results in low weight, calcium loss, and fragile bones (Lane et al., 2000). To assess the therapeutic effects of E.C.D. extracts *in vivo*, we inducted GIO in rats and processed E.C.D. extracts by gavage. Rats after GC administration gained less body weight (red line, square, Figure 1A) as weeks increased compared to rats in the Vehicle group (black line, circle), while rats that received E.C.D. extracts displayed increased body weight compared with rats in the GC group. With the dose of E.C.D. extracts increasing, the weight improvement efficacy is more dramatic. At week 8, rats receiving E.C.D. extracts of high dose (green line, diamond) gained more weight than rats in the GC group. Assessment of urine and serum revealed increased calcium and phosphorus loss in urine (Figures 1C,D) while decreased calcium and phosphorus levels in serum (Figures 1E,F) of rats in the GC group compared with rats in the Vehicle group, which was rescued when rats received E.C.D. extracts treatment. As the dose increased, rats restored the serum calcium and phosphorus concentration (Figures 1E,F). The level of calcium and phosphorus in urine (Figures 1C,D) was decreased by E.C.D. extracts of high dose. These data indicated that E.C.D. extracts can protect the loss of calcium and phosphorus and weight decrease in experimental GIO rats. The temperature (Figure 1B) of rats during the experiment fluctuated without significant differentiation.

### E.C.D. Extracts Restored the Bone Marrow Structure and Regulated Cytokine of Bone Resorption and Regeneration

**FIGURE 2:**
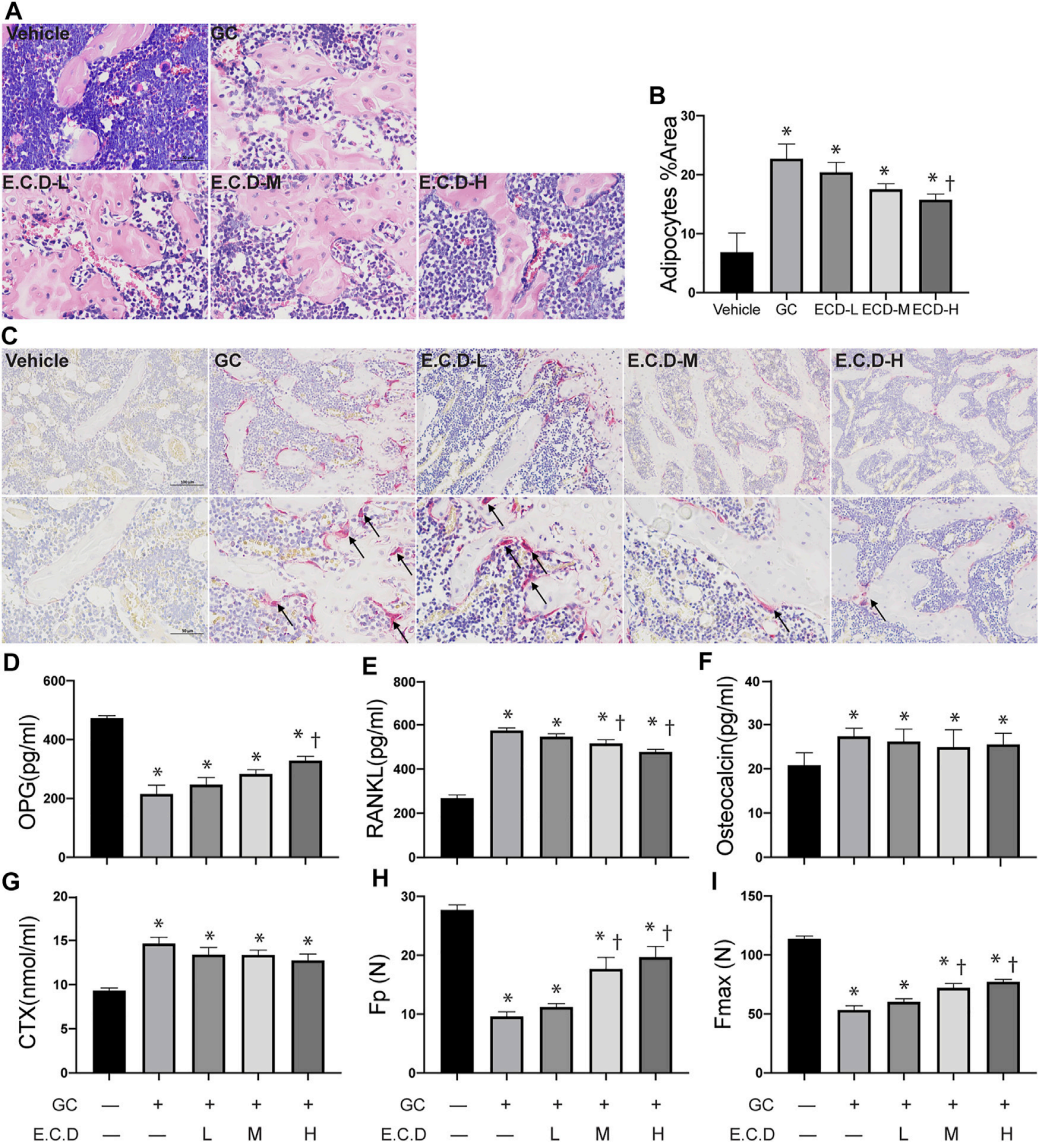
E.C.D. extracts restored the bone marrow structure and regulated cytokine of bone resorption and regeneration. **(A)** H&E staining was performed with femur of rats in five groups: Vehicle group (left upper panel), GC group (middle upper panel), B.C.D.–L group (left lower panel), B.C.D.–M group (middle lower panel), and B.C.D.–H group (right lower panel). **(B)** The quantitative adipocytes area in bone marrow for five groups, *n* = 3 in each group. **(C)** TRAP staining was assessed for five groups, upper panel is X200 magnification, lower panel is X400 magnification. The black arrow pointed to TRAP positive osteoclasts. **(D–G)** The OPG, RANKL, osteocalcin, and CTX level in rats’ serum were measured 8 weeks after GC administration, *n* = 8 in each group. **(H,I)** The Fp and Fmax of bone were assessed, *n* = 8 in each group. Data were analyzed as mean ± SEM, * vs. Vehicle group (GC–E.C.D.–), † vs. GC group (GC + E.C.D. –), *p* < 0.05.

The bone marrow deterioration was examined using H&E staining (Yang et al., 2020). In H&E staining (Figure 2A) the bone marrow cavity of the GC group was significantly enlarged with more light stained area composed by adipocytes compared with the Vehicle group, which was ameliorated by E.C.D. extracts treatment. The percentage of adipocytes area was quantified (Figure 2B) and E.C.D. extracts of high dose significantly decreased the adipocytes infiltration in bone marrow compared with the GC group. The TRAP staining (Bai et al., 2005) was performed to assess the activity of osteoclast (Figure 2C). After GC administration there were more osteoclast accumulation in trabeculae which was reversed by E.C.D. extracts treatment. The cytokine and chemokine in serum revealed the metabolism of osteoclast and osteoblast (Boyce and Xing, 2008). Bone remodeling is regulated by a crosstalk between bone-forming osteoblasts and bone-resorbing osteoclasts (Kim et al., 2020). OPG was synthesized by osteoblasts which inhibited osteoclast differentiation. The activation of RANKL-RANK signal could stimulate osteoclast formation. The OPG, RANKL, osteocalcin (an indicator of spontaneous bone loss) (Brown et al., 1984), and CTX (a bone resorption marker) (Iba et al., 2008) levels were measured by ELISA (Figures 2D–G). GC administration resulted in decreased OPG (related to osteoblast genesis) while increased RANKL, osteocalcin, and CTX (related to osteoclast genesis). Administration of E.C.D. extracts improved the OPG expression (E.C.D.-H group) and decreased RANKL expression (E.C.D.-M group and E.C.D.-H group) in serum compared with the GC group. But E.C.D. extracts did not decrease expression of osteocalcin and CTX in serum. The biomechanical function of bone is important to evaluate osteoporosis. The maximum load (Fmax) and elastic load (Fp) were dramatically decreased in rats of the GC group compared with the Vehicle group (Figures 2H,I). The E.C.D. extracts of medium and high dose treatment significantly increased the Fmax and Fp compared with the GC group.

### E.C.D. Extracts Improved the Integrity of Bone Structure and Increased Bone Density by Micro-CT Scan

**FIGURE 3:**
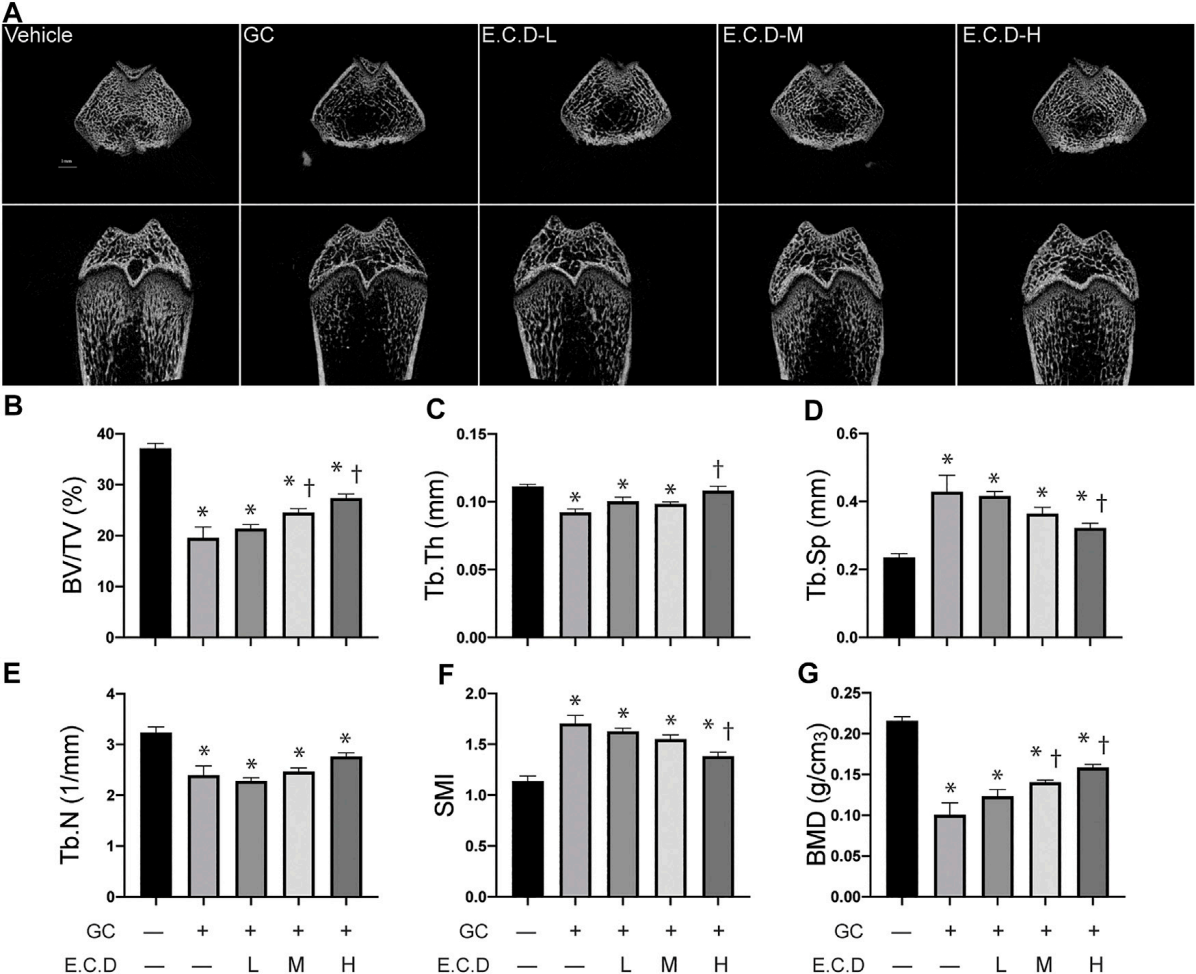
E.C.D. extracts improved the integrity of bone structure and increased bone density by micro-CT scan. **(A)** The micro-CT scan was processed to assess the micro-structure and the biomechanics of rats. The upper panel was the transverse section of the femur, and the lower panel was the longitudinal section of the femur. The scale bar was labeled as 1 mm. **(B–G)** Bone volume/total volume (BV/TV), trabecular thickness (Tb.Th), trabecular separation (Tb.Sp), trabecular number (Tb.N), structural model index (SMI), and bone mineral density (BMD) were measured 8 weeks after GC administration of rats. The columns from left to right are Vehicle group, GC group, B.C.D.–L group, B.C.D.–M group and B.C.D.–H group, respectively, *n* = 8 in each group. Data were analyzed as mean ± SEM, * vs. Vehicle group (GC–E.C.D.–), † vs. GC group (GC + E.C.D. –), *p* < 0.05.

3-D trabecular structural characteristics can help us understand the pathophysiology of osteoporosis. The compact trabecular bone structure was shown in the Vehicle group compared with decreased trabeculae volume and abnormal morphologies like scattered structure or fracture of the GC group (Figure 3A). E.C.D. extracts treatment improved the integrity and continuity of trabecular structure. Multi-angle and multi-section of bone structure was assessed by mico-CT and shown in Supplementary Figure S2. We further measured the bone volume/total volume (BV/TV), trabecular thickness (Tb.Th), and trabecular number (Tb.N) by micro-CT, which can reflect the trabecular bone volume, thickness, and number. GC administration remarkably decreased the volume of the trabecular, which reverted by E.C.D. extracts treatment in medium and high dose (Figure 3B). The number of trabecular also decreased in the GC group but E.C.D. extracts treatment did not improve it (Figure 3E). E.C.D. extracts also can improve trabecular thickness which was thinner in the GC group (Figure 3C). The trabecular separation (Tb.Sp) indicates the average width of the medullary cavity between the trabeculae, suggesting increased bone resorption. GC administration markedly increased Tb.Sp in rats compared with the Vehicle group (Figure 3D), while E.C.D. extracts (E.C.D.-H group) significantly reduced Tb.Sp compared with the GC group. The structural model index (SMI) reflects the rods and plates of trabecular. When osteoporosis occurs, the trabecular bone changes from a plate shape to a rod shape, resulting in SMI increasing. In our study, GC increased SMI (Figure 3F) compared with the Vehicle rats, which was decreased when the rats were treated with E.C.D. extracts (E.C.D.-H group). Bone mineral density (BMD) was dramatically reduced by GC administration compared with the Vehicle group, which was rescued by E.C.D. extracts (Figure 3G). With increased dosage, the BMD improved.

### E.C.D. Extracts Inhibited Osteoclast Differentiation *in vitro*

**FIGURE 4:**
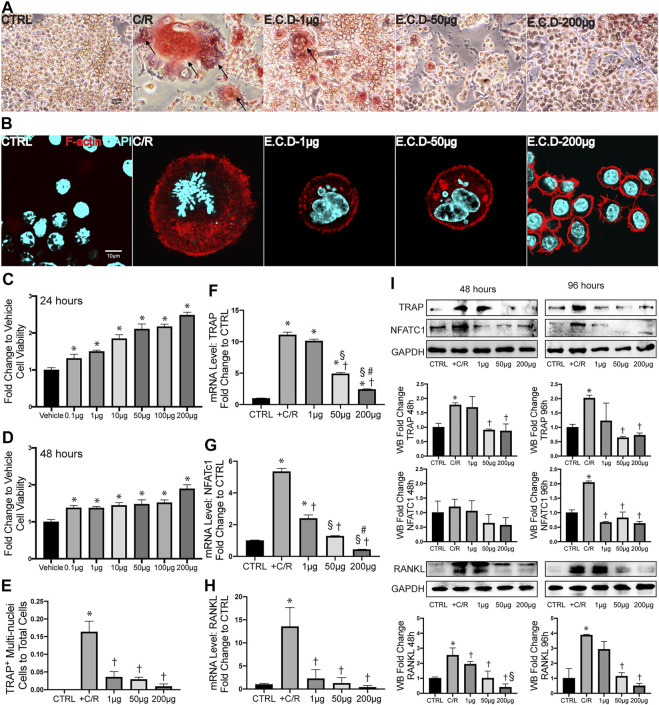
E.C.D. extracts inhibited osteoclast differentiation *in vitro*. **(A)** TRAP staining assay displayed TRAP positive cells in Raw264.7 cells at Day 5 after C/R stimulation. The black arrow points to a mature osteoclast. The scale bar is labeled as 20 μm. **(E)** The quantitative percentage of mature osteoclasts in total cells was assessed, *n* = 3 in each group. **(B)** F-actin staining was assessed at Day 5 after C/R induction. F-actin was stained ared color and DAPI was blue. The bright actin ring was formed in the C/R group, E.C.D.-L, and E.C.D.-M group. The scale bar is labeled as 10 μm. **(C,D)** To assess the toxicity of E.C.D. extracts to cells, we performed CCK8 assay *in vitro*. The Raw264.7 cells were seeded in 96-well plate (Vehicle group, first column from left) and the E.C.D. extracts in different doses were added (second column to seventh column from left). The CCK8 assay was measured 24 and 48 h later. *n* = 5 in each group. Data were analyzed as mean ± SEM, * vs. Vehicle group, *p* < 0.05. **(F–H)** The mRNA expression level of TRAP, NFATc1, and RANKL were measured 48 h after C/R stimulation, *n* = 3 in each group. **(I)** The protein level of TRAP, NFATc1, and RANKL were assessed at 48 and 96 h after C/R stimulation. The immunoblotting was shown in the upper panel and quantity of densitometric values fold change was shown in the lower panel. *n* = 3 in each group. Data were analyzed as mean ± SEM, * vs. CTRL group, † vs. + C/R group, § vs. 1 μg group, # vs. 50 μg group, *p* < 0.05.

The abnormal activation of osteoclast differentiation is one of the key reasons to induce osteoporosis. To first assess if E.C.D. extracts affect the growth properties of cells, we first processed the CCK8 assay. Interestingly, E.C.D. extracts improved cell viability and the efficacy enhanced as dose increased both in 24 and in 48 h without toxicity (Figures 4C,D). Next, the osteoclast differentiation was induced by adding C/R (50 ng/ml) to Raw264.7 cells. We evaluated TRAP staining and F-actin assay after 5 days. TRAP staining is used to stain tartrate-resistant acid phosphatase in osteoclasts. Raw264.7 cell is a small circle monocyte (left panel, Figure 4A). After C/R stimulation, Raw264.7 cell differentiated into osteoclast which displayed large and multinucleated morphology with TRAP positive staining (red color, left second panel). E.C.D. extracts inhibited osteoclast differentiation with less TRAP positive multinucleated cells (right three panel). The ratio of TRAP positive cells with ≥ 3 nuclei of total cells was quantified (Figure 4E). There are more TRAP positive cells after C/R stimulation compared with the CTRL group, and E.C.D. extracts significantly reduced the number of TRAP positive cells compared with the C/R group. Actin ring is a characteristic actin structure that is essential for bone resorption by osteoclasts. Differentiated osteoclasts after C/R stimulation showed bright circle red fluorescence Actin ring with multiple nuclei inside (left second panel, Figure 4B). Raw264.7 cells with E.C.D. extracts of 1 μg/ml and 50 μg/ml treatment also displayed Actin ring with multiple nuclei. E.C.D. extracts of 200 μg/ml reversed osteoclast differentiation (right panel) with no multinucleated osteoclast with F-actin positive staining. The bone resorption assay was also assessed (Supplementary Figure S3). Treatment of E.C.D. extracts was shown to reduce the area of resorption pit on bovine bone slices compared with the C/R group and the treatment function of E.C.D. extracts was dose-dependent that 200 μg/ml and 50 μg/ml E.C.D. extracts decreased more resorption area compared with E.C.D. extracts of 1 μg/ml. TRAP, NFATc1, and RANKL are important markers for osteoclast formation. We assessed these genes expression by qPCR. After C/R stimulation, the expression of TRAP, NFATc1, and RANKL were dramatically increased compared with the CTRL (Figures 4F–H). While these changes were reversed by E.C.D. extracts in three doses (1 μg/ml, 50 μg/ml, and 200 μg/ml). Moreover, as dose increased, the functional efficacy of E.C.D. extracts in inhibiting osteoclast differentiation increased. To further verify the hypothesis, we harvested the protein from Raw264.7 cells in 48 and 96 h, then the immunoblotting was performed, using GAPDH expression for normalization (Figure 4I). The expression of TRAP was increased in 48 and 96 h after C/R stimulation, which was reverted by E.C.D. extracts of 50 μg/ml and 200 μg/ml. C/R stimulation also increased NFATc1 expression in 96 h compared with CTRL (Raw264.7 cells without C/R stimulation), which were eliminated by E.C.D. extracts administration in doses of 1 μg/ml, 50 μg/ml, and 200 μg/ml. The expression of RANKL was increased in the C/R group both in 48 and 96 h compared with the CTRL group, and E.C.D. extracts of 50 μg/ml and 200 μg/ml significantly decreased it compared with the C/R group. These immunoblotting data indicated that E.C.D. extracts contributed to the inhibition of osteoclast differentiation, and the function is better in high doses (50 μg/ml and 200 μg/ml) than low doses (1 μg/ml).

### E.C.D. Extracts Promoted OPG Expression of MSC and Regulated the Paracrine Effect of MSC to Inhibit Osteoclastogenesis of Raw264.7 Cells

**FIGURE 5:**
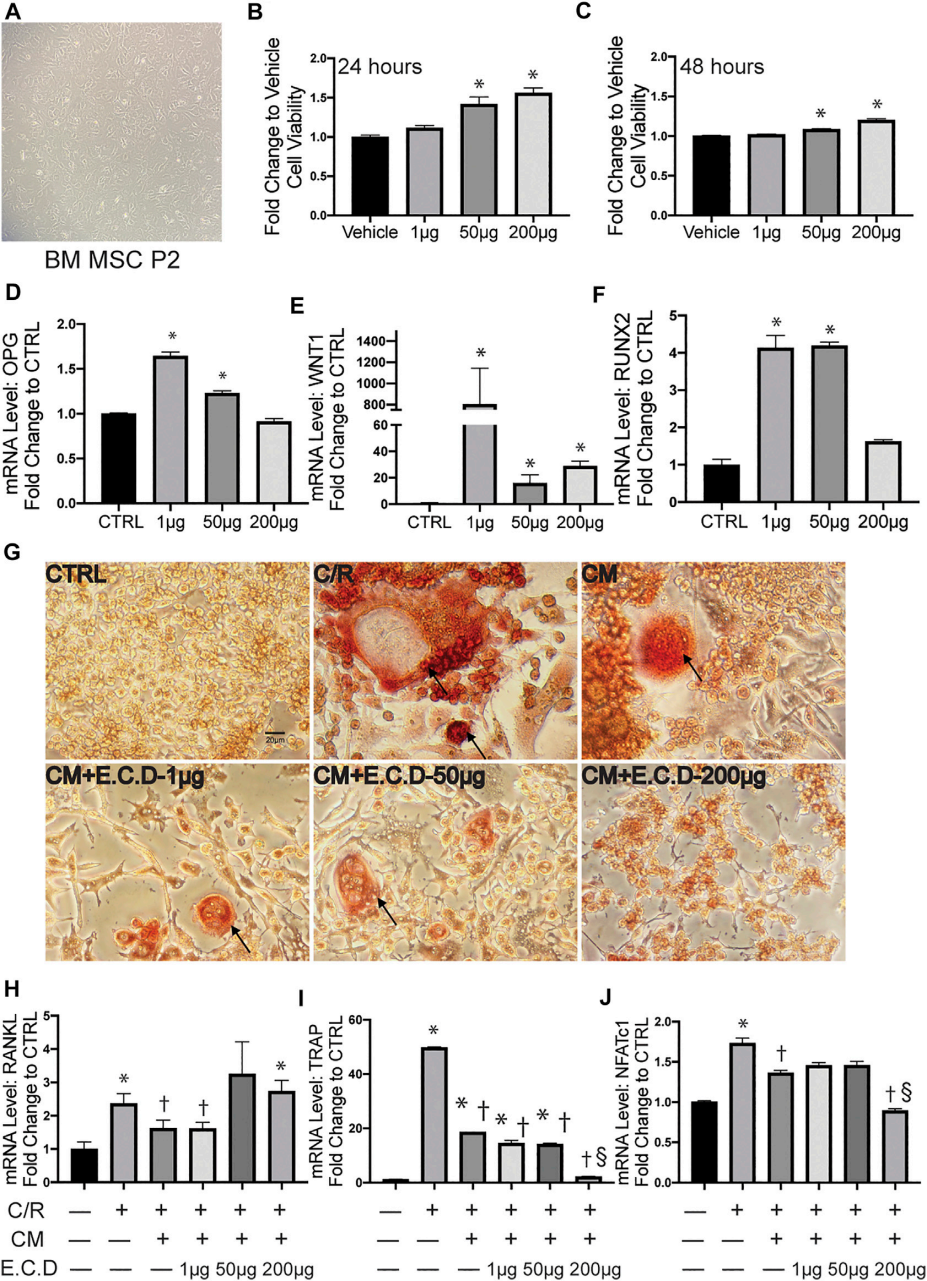
E.C.D. extracts promoted OPG expression of MSC and regulated paracrine effect of MSC to inhibit osteoclastogenesis of Raw264.7 cells. **(A)** The mice bone marrow MSC was harvested and cultured for expansion. **(B,C)** The toxicity of E.C.D. extracts to MSC was assessed by CCK8 assay. The MSCs were cultured in 96-well plate (Vehicle group), the E.C.D. extracts were added in different doses of 1 μg/ml, 50 μg/ml, and 200 μg/ml (1 μg, 50 μg, and 200 μg group, respectively). *n* = 5 in each group, data were analyzed as mean ± SEM, * vs. Vehicle group. **(D–F)** The mRNA level of OPG, Wnt1, and Runx2 were analyzed by adding different doses of E.C.D. extracts to MSC. *n* = 3 in each group. Data were analyzed as mean ± SEM, * vs. CTRL group, *p* < 0.05. (**G**) TRAP staining was assessed 5 days after C/R stimulation, the MSC CM, CM pretreated with E.C.D. extracts of 1 μg/ml, 50 μg/ml, and 200 μg/ml were added with C/R. The black arrow points to a mature osteoclast. The scale bar is labeled as 20 μm. **(H–J)** The paracrine function of MSC mediated by E.C.D. extracts was assessed. The MSC was treated with E.C.D. extracts and then the conditioned medium (CM) was harvested. The C/R stimulated Raw264.7 cells were treated with CM without being preconditioned with E.C.D. extracts, CM preconditioned with 1 μg/ml or 50 μg/ml or 200 μg/ml E.C.D. extracts. *n* = 3 in each group. Data were analyzed as mean ± SEM, * vs. CTRL group (first column from left), † vs. + C/R group (second column from left), § vs. CM group (third column from left). *p* < 0.05.

The bone metabolism is a cross-talking and dynamic process. After we proved the efficacy of E.C.D. extracts on inhibition of osteoclast differentiation, whether it plays a positive or a negative role on osteoblast genesis is also under investigation. Thus, we harvested MSC from mice bone marrow of tibia and femur (Figure 5A) and cultured them until passage 5. The CCK8 assay was assessed to clarify the function of E.C.D. extracts on MSC viability (Figures 5B,C). E.C.D. extracts of high dose (50 μg/ml and 200 μg/ml) improved MSCs viability on both 24 and 48 h. The OPG level and other genes related to osteoblast genesis were assessed by qPCR. Interestingly, the E.C.D. in low dose (1 μg/ml) dramatically increased OPG expression in MSCs (Figure 5D), while E.C.D. of 50 μg/ml still increased the OPG level but was a little bit lower than 1 μg/ml. As the dose increased to 200 μg/ml, the function of promoting OPG was lost (Figure 5D). The Wnt and Runx signaling are related to osteoblast formation (Komori, 2018; Komori, 2019; Karadeniz et al., 2020). Runx2 can induce Sp7, which is an essential transcription factor for osteoblast differentiation. For Wnt1, E.C.D. extracts in low dose (1 μg/ml) dramatically increased Wnt1 expression in MSC, E.C.D. extracts with 50 μg/ml and 200 μg/ml can increase Wnt1 level but lower than 1 μg/ml (Figure 5E). For Runx2, E.C.D. extracts with 1 μg/ml and 50 μg/ml significantly enhanced Runx2 level in MSC, but E.C.D. extracts of 200 μg/ml lost the function (Figure 5F). To further understand the regulating function of E.C.D. extracts on MSC paracrine effect in mediating osteoclast differentiation, we treated MSC with E.C.D. extracts for 48 h then harvested pre-conditioned medium (CM) of MSC. The CM of MSC was used in osteoclast differentiation assay, and TRAP staining was assessed (Figure 5G). Addition of C/R to cells greatly enhanced the number of TRAP positive cells, which was reversed by adding MSC CM. CM pretreated with E.C.D. extracts also decreased TRAP positive cells. The CM of MSC showed the function of decreasing NFATc1, TRAP, and RANKL expression of Raw264.7 cells (Figures 5H–J) compared with the C/R group. The CM of MSC pre-treated with E.C.D. extracts (200 μg/ml) decreased expression of TRAP and NFATc1 (Figures 5I,J) compared with the C/R group and better than the CM group (CM without re-treating with E.C.D. extracts). These data demonstrated that E.C.D. extracts also can inhibit osteoclast differentiation by regulating MSC’s paracrine effect. Interestingly, when we use CM of MSC pre-treated with E.C.D. extracts of high dose (200 μg/ml) treated Raw264.7 cells, the function of inhibiting RANKL expression is lost (Figure 5H).

### HPLC Analysis Identified Constituents of the E.C.D. Extracts

**FIGURE 6:**
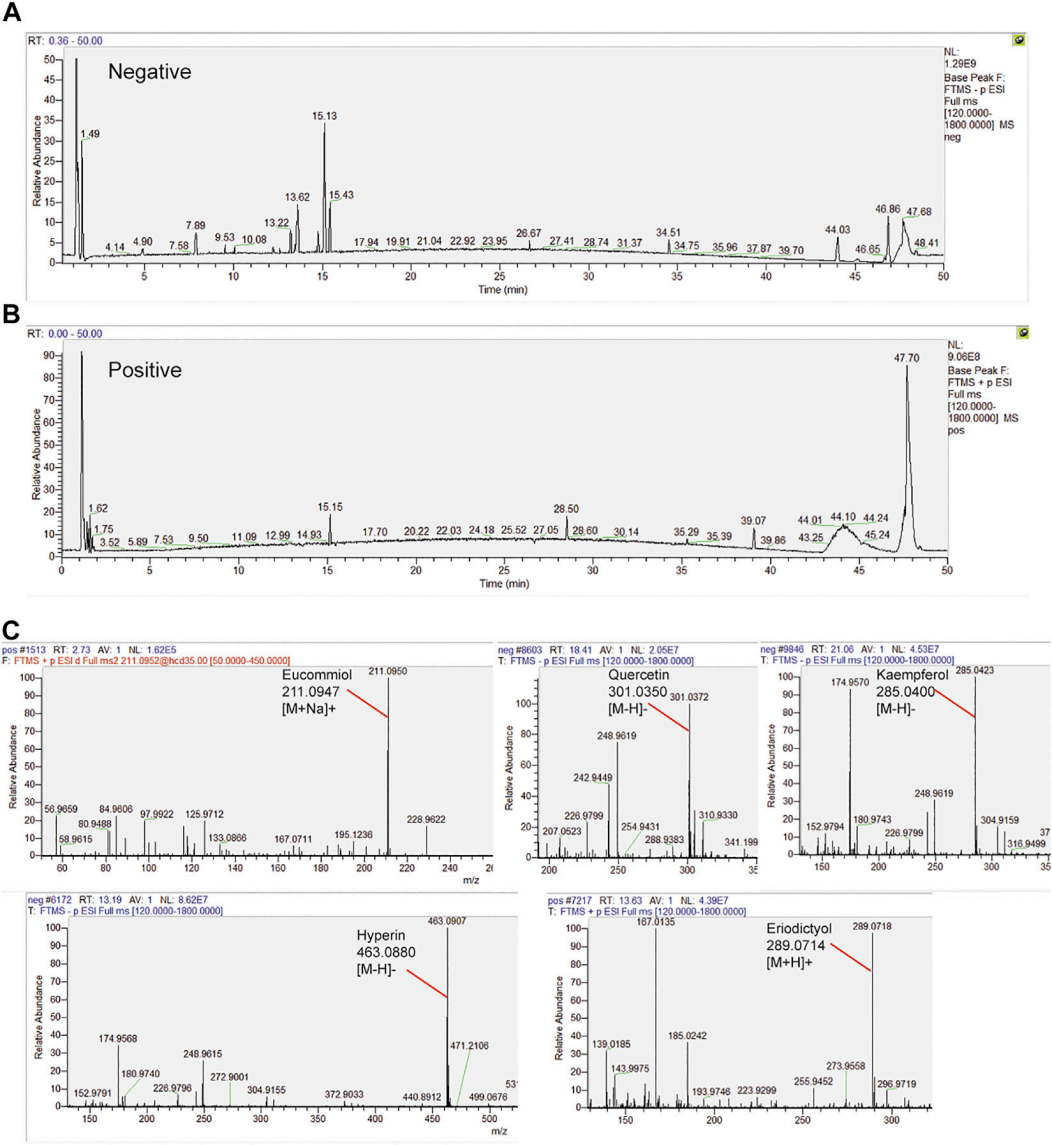
HPLC analysis identified constituents in the E.C.D. extracts. **(A,B)** Total ion chromatogram analyzed in positive and negative ion modes for E.C.D. extracts. **(C)** E.C.D. extracts constituent analysis: Eucommiol, Quercetin, Kaempferol, Hyperin, and Eriodictyol.

The samples were scanned in the negative and positive iron model (Figures 6A,B). The HPLC displayed strong signals, high peak capacity, and reproducibility for retention time. We collected a total of 250 constituents of E.C.D. based on the TCMSP database. Comparing with the molecular weight and structure of published constituents, we identified 72 constituents of E.C.D. extracts (47, 8, 23 from Eucommia, Cuscuta, and Drynaria respectively, 6 constituents exist in 2 herbs), including Eucommiol, Quercetin, Kaempferol, Hyperin, Eriodictyol (Figure 6C), and other constituents (shown in Supplementary Data Sheet S1).

### The Integrated Network Pharmacology Analysis Indicated the Potential Targets Mediated by E.C.D. Extracts in Treating Osteoporosis

**FIGURE 7:**
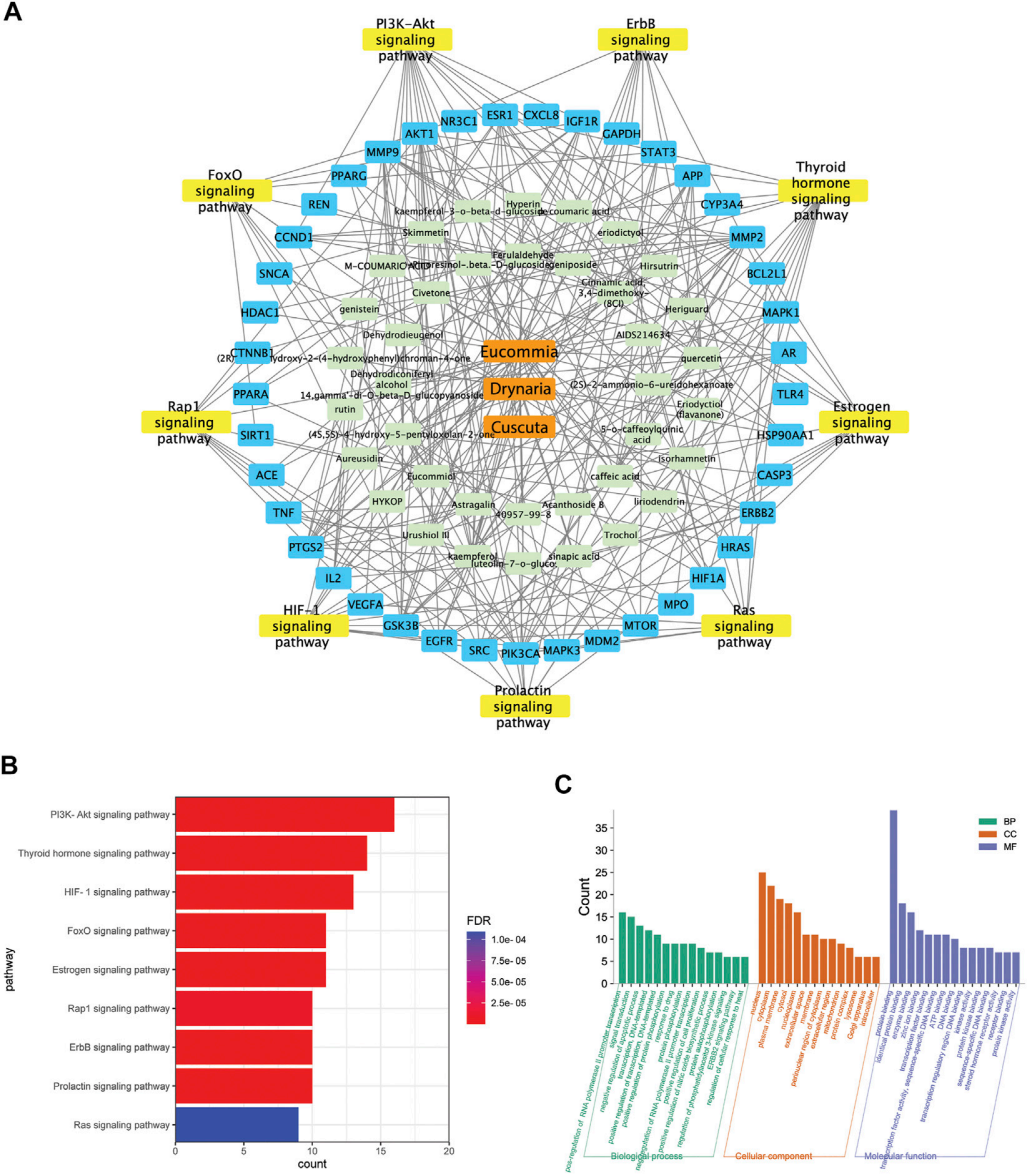
The integrated network pharmacology analysis indicated the potential targets mediated by E.C.D. extracts in treating osteoporosis. **(A)** E.C.D. extracts–constituent–osteoporosis related targets–pathway network. **(B)** The potential pathways in treating osteoporosis by E.C.D. extracts performed by KEGG enrichment analysis. *p* < 0.05, the count of enrichment related targets decreases from top to bottom. **(C)** The biological process (BP), molecular function (MF), and cellular components (CC) analysis of targets mediated by E.C.D. extracts. *p* < 0.05, the count of related targets decreases from left to right.

The targets mediated by identified constituents of E.C.D. extracts were analyzed by Swiss Target Prediction. Meanwhile, we collected 4576 targets related to osteoporosis from DrugBank database, OMIM database, and GeneCards database. Comparing with published information of osteoporosis related targets, 44 targets mediated by the constituents of E.C.D. extracts had been identified with the potential of treating osteoporosis (Figure 7A). The 44 targets were mapped to a total of 101 pathways using the DAVID database. Unrelated pathways, such as “Pathways in cancer” were excluded. The pathways that satisfied the criteria (*p*-value <0.05, count>8, removing other disease-related pathways) were listed (Figure7B). Herbs-constituents-targets osteoporosis-related pathway network was constructed (Figure 7A). Three components of E.C.D. extracts related to 38 constituents, mediating 41 osteoporosis-related targets. The top nine pathways with the largest number of enrichment targets were considered as the main biological processes involved in osteoporosis treatment. They are the PI3K/Akt pathway, thyroid hormone pathway, HIF-1 pathway, FoxO pathway, estrogen pathway, Rap1 pathway, ErbB pathway, prolactin pathway, and Ras pathway. We next elucidate the biological process (BP), molecular function (MF), and cellular components (CC) of E.C.D. extracts involved in Figure 7C. For BP analysis, the main hubs play an important role on upregulating cell proliferation and downregulating cell apoptosis, positively regulating protein phosphorylation and PI3K signaling pathway. The main molecular function related to the targets are protein binding, enzyme binding, zinc ion binding, and transcription factor binding. Collecting KEGG pathway analysis, BP, MF, and CC analysis together, these data indicated E.C.D. extracts may exert therapeutic effect on osteoporosis through PI3K/Akt signaling pathway.

### E.C.D. Extracts Inhibited Osteoclast Differentiation Through Regulating PI3k/Akt Pathway

**FIGURE 8:**
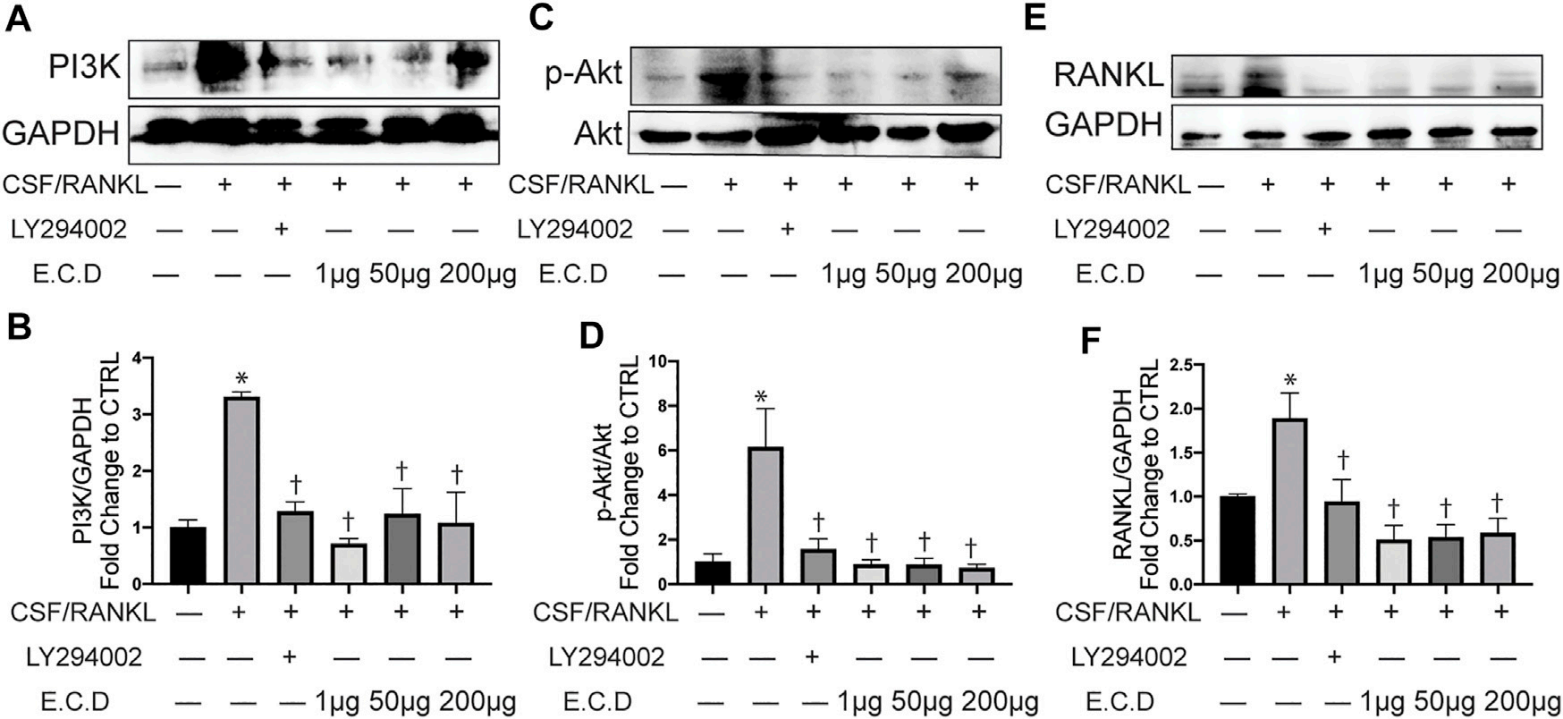
E.C.D. extracts inhibited osteoclast differentiation through regulating PI3k/Akt pathway. PI3K inhibitor (LY294002) was added 2 h before C/R induction. Raw264.7 cells were stimulated with C/R. E.C.D. extracts in different doses (1 μg/ml, 50 μg/ml, 200 μg/ml) were added at the same time for 48 h. **(A)** Representative Western blotting images of PI3K and GAPDH. **(B)** Fold changes in relative densitometric values of PI3K. **(C)** Representative Western blotting images of p-Akt and Akt. **(D)** Fold changes in relative densitometric values of p-Akt. **(E)** Representative Western blotting images of RANKL and GAPDH. **(F)** Fold changes in relative densitometric values of RANKL. *n* = 3, data was analyzed as mean ± SEM, * vs. CTRL group (CSF/RANKL–LY294002– E.C.D–), † vs. C/R group (CSF/RANKL + LY294002–E.C.D–), *p* < 0.05.

The potential targets and pathways of E.C.D. extracts in mediating GIO were further verified by Western blot *in vitro*. PI3K is a protein usually promoting cell survival and proliferation. In many diseases, PI3K/Akt pathway regulates the abnormal stimulation of biological activities to induce a pathology process. In osteoporosis, a published paper showed abnormal activity of PI3K/Akt pathway can stimulate osteoclast differentiation (Lukert and Raisz, 1990). Raw264.7 cells expressed more PI3K after C/R stimulation (Figure 8A), while E.C.D. extracts down regulated the expression of PI3K. The quantitative bar graph (Figure 8B) of PI3K indicated that both 1 μg/ml, 50 μg/ml, and 200 μg/ml E.C.D. extracts can significantly inhibit PI3K expression. C/R induction also increased phosphorated Akt (Figure 8C), which was reversed by E.C.D. extracts administration in three doses (Figure 8D). To further verify that the PI3K/Akt pathway regulates osteoclast differentiation, we added one group with PI3K inhibitor (LY294002) (third column from left, Figures 8A,C,E). LY294002 significantly inhibited PI3K expression (Figure 8B) and it also decreased phosphorated Akt (Figure 8D). We further assessed the RANKL expression regulated by LY294002. The Western blot result indicated that the level of RANKL was down regulated by the LY294002 (Figure 8E). The P38(MAPK) was also assessed as another signaling protein to promote osteoclast differentiation. After C/R stimulation, Raw264.7 cells expressed more p-P38 (phosphorated P38) compared with CTRL (Supplementary Figure S4E). E.C.D. extracts decreased p-P38 expression compared with the C/R group with significance in 50 μg/ml and 200 μg/ml (Supplementary Figure S4F). These data indicate that E.C.D. extracts inhibit osteoclast differentiation through regulating PI3K/Akt pathway and have the potential to regulate MAPK.

## Discussion

Glucocorticoid-induced osteoporosis (GIO) is the most common secondary cause of osteoporosis which results in server morbidity (Compston, 2018). Glucocorticoids can increase the production of M-CSF and RANKL which promote osteoclast differentiation and decrease production of osteoprotegerin (OPG) by osteoblastic cells (Swanson et al., 2006). Even though there are several choices for osteoporosis management, the clinical therapy remains suboptimal. For osteoporosis treatment, estrogen is selected for the treatment of OP patients with obvious menopausal symptoms and long-term use is not recommended (Fait, 2019). Bisphosphonates are currently the most commonly used medication for osteoporosis treatment such as alendronate, risedronate, and zoledronic acid (Qaseem et al., 2017). However, it has the risk of osteonecrosis of the jaw and atypical femoral fracture (Adler et al., 2016). Denosumab is also used as the first recommended approval drug to prevent osteoporosis. It works as a RANK binding inhibitor to reduce bone resorption (Miller, 2009). However, the clinical study found discontinuation of denosumab can cause rapid bone loss and increase the risk of multiple rebound-related vertebral fractures (RAVF) (Anastasilakis et al., 2021). The ideal strategy to treat osteoporosis is to both inhibit osteoclast and promote osteoblast formation. Existing treatments mostly focus on inhibiting bone desorption with side effects for long-term use.

The use of TCM as a potential treatment strategy for osteoporosis is an evolving field of investigation. Published literature and clinical trials provided evidence of efficacy of TCM herbs in experimental and clinical osteoporosis treatment (An et al., 2016; Wang et al., 2017). However, there is still a lack of strong experimental evidence and mechanism elaboration of TCM treatment. More effective therapy is under investigation. In the present study, we elaborated the efficacy of E.C.D. extracts in treating experimental GIO rats. GIO results in an overactive osteoclast and bone resorption in rats, with low levels of calcium and phosphorus in serum. The calcium and phosphorus were lost from urine which results in a decreased level in serum. Moreover, the GC administration resulted in weight decrease of rats. In our study, E.C.D. extracts treatment protected the weight loss of rats after GC administration. With the doses increasing, the protecting function improved. Meanwhile, the level of calcium and phosphorus in serum were improved by E.C.D. extracts compared with the GC group. The dose of E.C.D. extracts effected the therapeutic function. With the dose increasing, E.C.D. extracts are more effective. Apart from protecting loss of serum calcium and phosphorus, the cytokines in serum also reflected the bone metabolism balance. E.C.D. extracts with high dose significantly decreased the osteoclast-related cytokine release RANKL and increased the osteoblast-related cytokine OPG. The bone histology also showed the trabecular bone destruction and bone marrow loss in the GC group. E.C.D. extracts effectively improved the bone structural elasticity and hardness of GIO rats based on the bone biomechanical measurement. Bone strength depends on all three factors: bone geometry, thickness, and density (Buehring et al., 2013). The micro-CT scan helped to further understand the effect of E.C.D. extracts on bone microstructure and bone strength of experimental GIO rats, which indicated E.C.D. extracts dramatically improved the integrity, thickness, and density of bone in GIO rats.

Currently, most treatments in clinic for osteoporosis are phosphate supplements or targeting through regulating bone metabolism by RANKL/OPG pathway (Compston et al., 2019). MAPK/NF-κB and Wnt/GSK-3β/β-catenin signaling pathway are also demonstrated to play important roles in osteoclast genesis (An et al., 2019; Wang et al., 2020). Xiao L. et al. found Puerarin can suppress osteoclastogenesis via inhibition of TRAF6/ROS-dependent MAPK/NF-κB signaling pathways (Masanobu, 2014) in ovariectomy (OVX)-induced osteoporosis model mice. Even though several signaling pathways are shown as a critical effector for osteoclast formation, there is still under investigation the mediation of TCM to potential signaling pathway during osteoclast formation. The natural herbs are usually more difficult to be clarified in a specific pathway which regulates biological metabolism because of its complex components especially for compound. To further understand the mechanism of the function of E.C.D. extracts alleviating osteoporosis, we processed HPLC analysis and investigated the potential targets mediated by E.C.D. extracts which have the potential to treat osteoporosis. The potential targets of E.C.D. extracts which may mediate the signaling pathway to treat osteoporosis were analyzed by the network pharmacology analysis. KEGG enriched pathway indicated E.C.D. extracts have the identified constituents which mostly targeted proteins in the PI3K/Akt pathway. The PI3K/Akt pathway is a crucial signaling pathway regulating cell proliferation and survival. Jang Bae Moon et al. demonstrated PI3K/Akt/GSK3β/NFATc1 signaling pathway regulates osteoclast differentiation (Moon et al., 2012). Wu L et al. found PI3K/Akt/Blimp1 signaling pathway promotes osteoclast formation (Wu et al., 2017). Tsubaki et al. (2014) found inhibition of PI3K/Akt pathways can suppress osteoclast formation. Activation of PI3K/Akt signaling cascade plays a crucial role in hyperactivation of osteoclasts in osteoporosis. For our study, to verify our hypothesis, we used PI3K inhibitor (LY294002) to pretreat Raw264.7 cells before C/R stimulation. The Western blot results indicated E.C.D. extracts inhibited PI3K expression and phosphorated Akt. At the same time, inhibition of the PI3K/Akt pathway decreased RANKL expression. Therefore, we believe E.C.D. extracts exert function of inhibiting osteoclastogenesis through regulating the PI3K/Akt signaling pathway.

Finally, our experiment also has limitations. We demonstrated that E.C.D. extracts can alleviate experimental GIO *in vivo* and inhibit osteoclast differentiation through regulating the PI3K/Akt pathway *in vitro*. However, the bone metabolism depends on two biological processes: bone resorption by osteoclast and bone formation of osteoblast. We found E.C.D. extracts increased the OPG expression *in vivo* and promoted MSC proliferation and expression of OPG, Wnt1, and Runx2 (the protein related to osteoblast formation) *in vitro*. However, more experiments need to be conducted in the future to demonstrate this hypothesis. Also, based on a published paper, the PI3K/Akt pathway also regulates osteoblast genesis, the mechanism of E.C.D. extracts in mediating osteoblast by the PI3K/Akt pathway still need to be clarified.

The combination of Eucommia, Cuscuta, and Drynaria is widely used in TCM decoction for osteoporosis treatment. However, there are rare studies investigating the osteoporosis treatment efficacy of the combination and further clarify the mechanism. Our work proved that E.C.D. extracts have the significant efficacy in treating experimental GIO rats *in vivo*. Moreover, we processed a series of experiments *in vitro* to clarify the potential biological mechanism. Our work suggested E.C.D. extracts can down-regulate PI3K/Akt pathway to inhibit osteoclastogenesis and have therapeutic efficacy in GIO rats. It provides a novel perspective for GIO treatment in the future.

## Abbreviation

GC, glucocorticoid; GIO, glucocorticoid-induced osteoporosis; TCM, traditional Chinese medicine; MSC, mesenchymal stromal cell; FBS, fetal bovine serum; P.S.G, penicillin/streptomycin/L-glutamine; E.C.D., Eucommia, Cuscuta, Drynaria; RANKL, Receptor Activator of NF-κB Ligand; OPG, osteoprotegerin; CTX, type I collagen; Fmax, maximum load; Fp, elastic load; micro CT, micro computed tomography; BMD, bone mineral density; BV/TV, ratio of trabecular bone volume to total volume; Tb.Th, mean thickness of bone trabecular; Tb. Sp, trabecular spacing; Tb.N, the number of bone trabeculae per millimeter distance; SMI, structural model index; M-CSF, macrophage colony stimulating factor; PI3K, phosphatidylinositol 3 kinase; Akt, protein kinase B; TRAP, tartrate-resistant acid phosphate.

## Data Availability

The original contributions presented in the study are included in the article/Supplementary Material. Further inquiries can be directed to the corresponding authors.

## Supplementary Material

Supplementary Figure S1
**(A)**Fingerprint of Eucommia extract. **(B)** Fingerprint of Cuscuta extract. **(C)** Fingerprint of Drynaria extract.

Supplementary Figure S2 Micro-CT images of femur of GIO rats.

Supplementary Figure S3 E.C.D. extracts reduced the resorption area on bone slices. **(A)** Representative phase contract images of bone resorption pit. The black arrow pointed to the resorption pit. **(B)** Representative images of toluidine blue staining of bone resorption pit. The black arrow pointed to dark blue staining of the resorption pit. **(C)** The percentage area of bone resorption pit was assessed by Image J. *n* = 3 in each group. Data was analyzed as mean ± SEM, * vs. CTRL group, † vs. +C/R group, P<0.05.

Supplementary Figure S4
**(A,C,E)** Western blot images of PI3K, GAPDH, p-Akt, Akt, p-P38, and P38. **(B,D,F)** Quantitative protein expression of PI3K, p-Akt, and p-P38. *n* = 3 in each group. Data was analyzed as mean ± SEM, * vs. CTRL group, † vs. +C/R group, P<0.05.
