# Supplementary material for: Eucommia, Cuscuta, and Drynaria Extracts Ameliorate Glucocorticoid-Induced Osteoporosis by Inhibiting Osteoclastogenesis Through PI3K/Akt Pathway
Source: Front Pharmacol. 2022 Feb 4;12:772944. doi: 10.3389/fphar.2021.772944 (PMC8855109; doi:10.3389/fphar.2021.772944)
Supplement: Supplementary file 1 [file DataSheet2.docx]

**Table S1**

**Antibodies for Western Blot Analysis**

| **Target** | **Company** | **Catalog Number** | **Isotype** | **Host Species** |
| --- | --- | --- | --- | --- |
| RANKL | Proteintech | 23408-1-AP | IgG | Rabbit |
| NFATC1 | abcam | ab25916 | IgG | Rabbit |
| TRAP | Proteintech | 16668-1-AP | IgG | Rabbit |
| PI3K | Proteintech | 67071-1-Ig | IgG2b | Mouse |
| p-Akt | abcam | ab81283 | IgG | Rabbit |
| Akt | abcam | ab179463 | IgG | Rabbit |
| p-P38 | abcam | ab195049 | IgG | Rabbit |
| P38 | abcam | ab47363 | IgG | Rabbit |
| GAPDH | abcam | ab8245 | IgG1 | Mouse |

**Table S2**

**ELISA Kit for Cytokine Analysis of Serum**

| **Target** | **ELISA Kit** | **Company** | **Catalog Number** |
| --- | --- | --- | --- |
| BGP | Rat Undercarboxylated Osteocalcin ELISA Kit | R&D | NBP2-79682 |
| NTX | Rat NTX1 ELISA Kit | R&D | NBP2-76473 |
| OPG | Rat Osteoprotegerin/TNFRSF11B ELISA Kit | R&D | NBP2-76685 |
| RANKL | Rat RANKL ELISA Kit | Biorbyt | orb385429 |
